# SNP Alleles Associated With Low Bolting Tendency in Sugar Beet

**DOI:** 10.3389/fpls.2021.693285

**Published:** 2021-07-12

**Authors:** Samathmika Ravi, Giovanni Campagna, Maria Cristina Della Lucia, Chiara Broccanello, Giovanni Bertoldo, Claudia Chiodi, Laura Maretto, Matteo Moro, Azam Sadat Eslami, Subhashini Srinivasan, Andrea Squartini, Giuseppe Concheri, Piergiorgio Stevanato

**Affiliations:** ^1^Department of Agronomy, Food, Natural Resources, Animals and Environment, University of Padova, Legnaro, Italy; ^2^Cooperativa Produttori Agricoli Società Cooperativa Agricola (COPROB), Minerbio, Italy; ^3^Institute of Bioinformatics and Applied Biotechnology, Bengaluru, India

**Keywords:** sugar beet, autumnal sowing, vernalization, bolting tolerance, SNP detection technologies, SNP association, marker-assisted breeding

## Abstract

The identification of efficient molecular markers related to low bolting tendency is a priority in sugar beet (*Beta vulgaris* L.) breeding. This study aimed to identify SNP markers associated with low bolting tendency by establishing a genome-wide association study. An elaborate 3-year field trial comprising 13 sugar beet lines identified L14 as the one exhibiting the lowest bolting tendency along with an increased survival rate after autumnal sowing. For SNP discovery following phenotyping, contrasting phenotypes of 24 non-bolting and 15 bolting plants of the L14 line were sequenced by restriction site-associated DNA sequencing (RAD-seq). An association model was established with a set of 10,924 RAD-based single nucleotide polymorphism (SNP) markers. The allelic status of the most significantly associated SNPs ranked based on their differential allelic status between contrasting phenotypes (*p* < 0.01) was confirmed on three different validation datasets comprising diverse sugar beet lines and varieties adopting a range of SNP detection technologies. This study has led to the identification of SNP_36780842 and SNP_48607347 linked to low bolting tendency and can be used for marker-assisted breeding and selection in sugar beet.

## Introduction

Sugar beet is an important crop in the temperate areas providing about 30% of total sugar (OECD-FAO, 2020). In the global change scenario, an emerging priority for sugar beet breeding is to increase the yield in the face of recurrent and adverse environmental conditions, such as cold, thermal, water, and nutritional stresses (Hoffmann and Kenter, 2018; Abou-Elwafa et al., 2020).

One of the ways to increase sugar beet yield is by taking advantage of different cultivation patterns. Biennial beets, grown for their sugar, are conventionally sown in the spring in temperate climates and harvested in summer–autumn. To avoid drought and thermal stresses late in the season, the practice of early sowing and/or autumnal sowing of sugar beet is weighed fundamental for increasing sugar yield (Hoffmann and Kluge-Severin, 2011; Schnepel and Hoffmann, 2016; Hoffmann and Kenter, 2018; Höft et al., 2018a,b). While widening the cultivation period has several advantages, the problem of premature bolting becomes more pertinent in the early sowing and winter breeding of beets.

Bolting or elongation of the stem followed by flowering under certain environmental conditions (e.g., variation in day length, changes in temperature, water stress, and/or hormone imbalance) affects different plant species, such as sugar beet (Dally et al., 2018; Höft et al., 2018a). During this vegetative to reproductive transition, most resources of plants are diverted away from the vegetative parts, rendering them not only inedible by affecting the sugar content but also affecting the harvest (Nelson and Deming, 1952; Scott et al., 1973; Lasa, 1977). In addition to this, the fertile seeds produced have high disseminating potential. Due to the above-mentioned undesirable consequences, the control of bolting is crucial (Mutasa-Göttgens et al., 2010; Kuroda et al., 2019). Several mechanical, electrical, and chemical methods for bolting control have been proposed and experimented with, but studies report them as cost-prohibitive in large breeding programs (Diprose et al., 1985; Ferrandino et al., 2008).

The genetic basis and improvement of bolting control in sugar beet have been well-studied (Dally et al., 2014). The annual habit in *B. vulgaris* was shown to be controlled by a major dominant gene, commonly referred to as the bolting gene *B*, which promotes the initiation of bolting under long days without prior vernalization (Abou-Elwafa et al., 2012). Although annual beets that are heterozygous at the *B* locus (*Bb*) bolt readily without vernalization, heterozygotes derived from crosses between annual and biennial beets exhibit a delay in bolting compared with annual (*BB*) beets (Mutasa-Göttgens et al., 2010; Abou-Elwafa et al., 2012). In a study with three F_3_ mapping populations, it was demonstrated that the vernalization requirement co-segregated with the bolting locus *B* on chromosome 2, and seasonal bolting was controlled by two major quantitative trait loci (QTLs) located in chromosomes 4 and 9 of sugar beet. In the same study, it was shown that late seasonal bolting alleles, SBT_4 and SBT-9 (BR1) in combination with biennial alleles at B might provide beet genotypes with bolting resistance (Tränkner et al., 2017). The haplotype sequences of important flowering genes, such as *BTC1, BvBBX19, BvFT1*, and *BvFT2* in sugar beet have also been exploited to understand their adaptation and cultivation of biennial beets in the context of bolting resistance (Höft et al., 2018a,b). Literature also shows that few major loci enrich the variation of bolting time in beet and that the identification of further alleles will support the development of beet genotypes with purpose-oriented bolting time (Broccanello et al., 2015; Stevanato and Biscarini, 2016; Tränkner et al., 2016; Pfeiffer et al., 2017). The genetic analysis of bolting phenotypes has used a variety of DNA markers for the detection of QTLs controlling bolting (Hébrard et al., 2016; Tränkner et al., 2016; Pfeiffer et al., 2017; Kuroda et al., 2019). These have been primarily performed on mortal mapping populations, i.e., either F_2_ or F_3_, and the identified bolting linked markers may segregate as a consequence of crossing over between markers and QTLs in subsequent generations. These methods are often laborious and limited by the density of markers. Single nucleotide polymorphisms or SNPs are attractive because of their large presence in the genome and ease of detection, allowing high throughput use in marker-assisted breeding and selection in various crops (Agarwal et al., 2008; McCouch et al., 2010; Choi et al., 2020).

However, the identification of novel SNP markers to efficiently control bolting is still a key challenge.

In this study, SNP markers associated with bolting tolerance post-winter and vernalization were identified by (a) phenotyping 13 sugar beet lines for bolting tolerance after autumnal sowing from a 3-year field trial, (b) RAD-sequencing of the line L14 combining highest survival and the lowest bolting percentage, (c) establishing a Genome-wide association study (GWAS) to identify favorable alleles related to low bolting tendency, and (d) validating the identified SNP markers using a range of SNP genotyping methods on diverse sugar beet lines and varieties. The results from this study precede controlled bolting of sugar beet breeding through marker-assisted selection and provides insights into candidate genes involved in the complex process of flowering and cold tolerance in sugar beet.

## Results

### Bolting and Survival Rates of 13 Assessed Sugar Beet Lines

**Figure 1:**
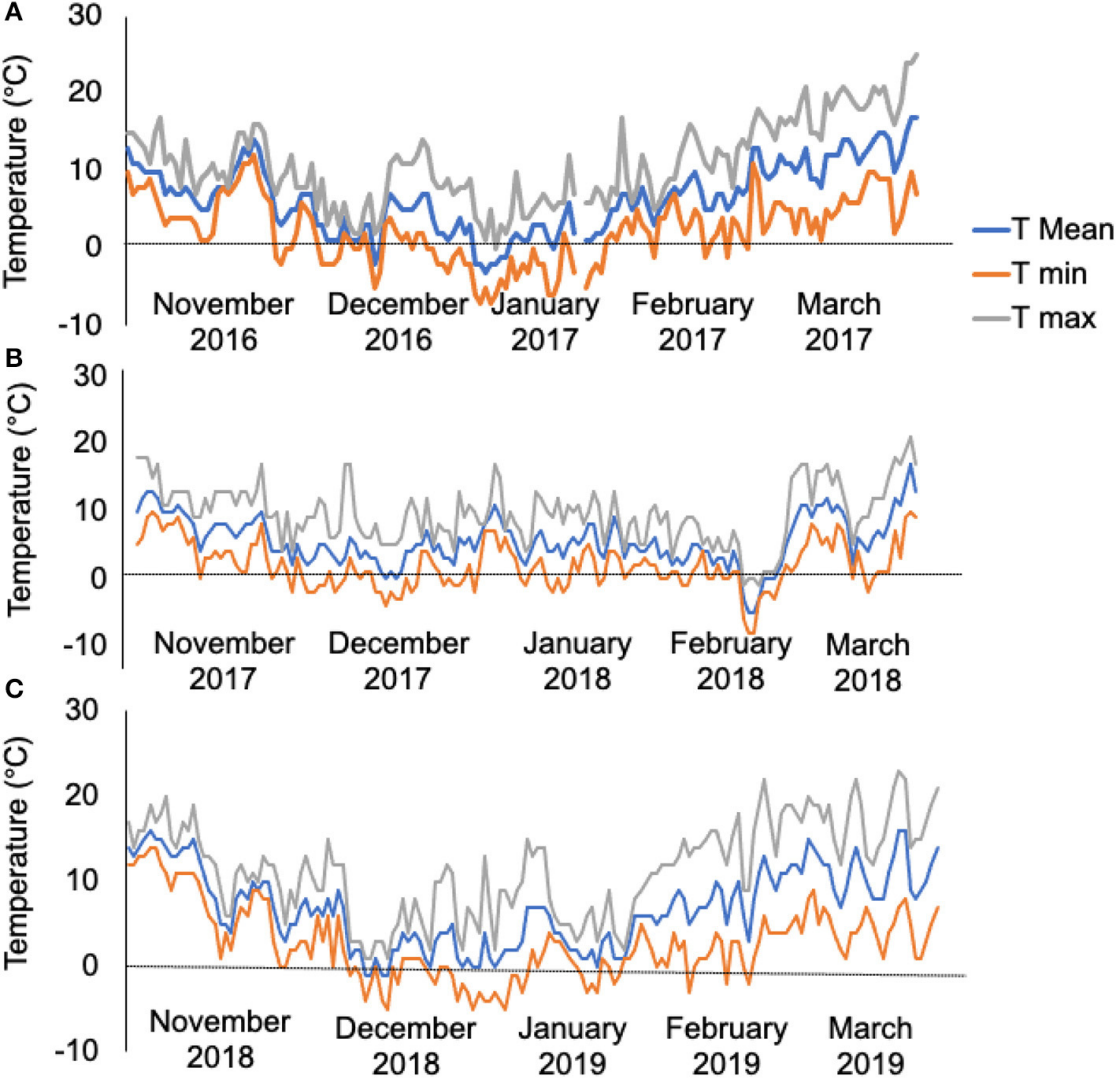
**(A)** Temperature profiles as recorded from the field during 2016–2017, 50 days of temperatures < 0°C. **(B)** Temperature profiles as recorded from the field during 2017–2018, 35 days of temperatures < 0°C. **(C)** Temperature profiles as recorded from the field during 2018–2019, 33 days of temperature < 0°C. The trend line in gray, orange, and blue represents maximum temperature (T max), minimum temperature (T min), and mean temperature (T mean) per day of the month, respectively.

The meteorology data revealed that the experimental site experienced 50 days of temperatures < 0°C from November 2016 to March 2017 (Figure 1A, 1st-year field trial), 35 days of temperatures < 0°C from November 2017 to March 2018 (Figure 1B, 2nd-year field trial), and 33 days of temperatures < 0°C from November 2018 to March 2019 (Figure 1C, 3rd-year field trial). The contrast in the bolter count and survival rates among the sugar beet genotypes are shown in Table 1. It was observed that individuals of the L14, L8, and L3 lines presented a lower percentage of bolters along with higher survival rates post-winter. Although the lines L5 and L13 showed a lower bolter percentage, they had a very low survival rate post-winter of <10% of the initial plants. The remaining lines L12, L6, L1, L4, L7, L11, L9, and L2 combined phenotypes of high bolting percentage along with lower survival rates post-winter. Subsequently, the field trial resulted in the identification of a line, L14, combining characteristics of least bolting tendency and highest survival rate after autumnal sowing. Additionally, we genotyped the material with the SNP marker, *SNP183*, linked to bolting tolerance generated in an earlier effort by our lab (Broccanello et al., 2015). The genotyping results from *SNP183* are presented in Supplementary Table 1 and are suggestive of L14 having 95% frequency of the low bolting allele. L14 was, thus, considered as an ideal plant material for further sequencing and understanding the basis of bolting tolerance post vernalization.

**Table 1:** Mean values (and standard error) from the phenotyping of 13 sugar beet lines assessed for the number of bolting plants and the number of survived plants post-winter.

**Name**	**Number of individuals before winter**	**Number of plants that survived post winter**	**Number of bolters**
L14	750	296 ± 6	12 ± 2
L8	750	221 ± 9	26 ± 4
L3	750	219 ± 4	51 ± 3
L5	750	73 ± 12	6 ± 2
L13	750	67 ± 9	11 ± 3
L12	750	76 ± 11	42 ± 2
L6	750	64 ± 12	64 ± 2
L1	750	75 ± 5	75 ± 6
L4	750	77 ± 4	77 ± 2
L7	750	72 ± 3	72 ± 3
L11	750	70 ± 4	70 ± 5
L9	750	71 ± 8	71 ± 3
L2	750	67 ± 6	67 ± 4

*L14 was identified as the line combining the highest survival percentage with the lowest bolting percentage post-winter*.

### Analysis of the Genetic Structure of L14 Line

**Figure 2:**
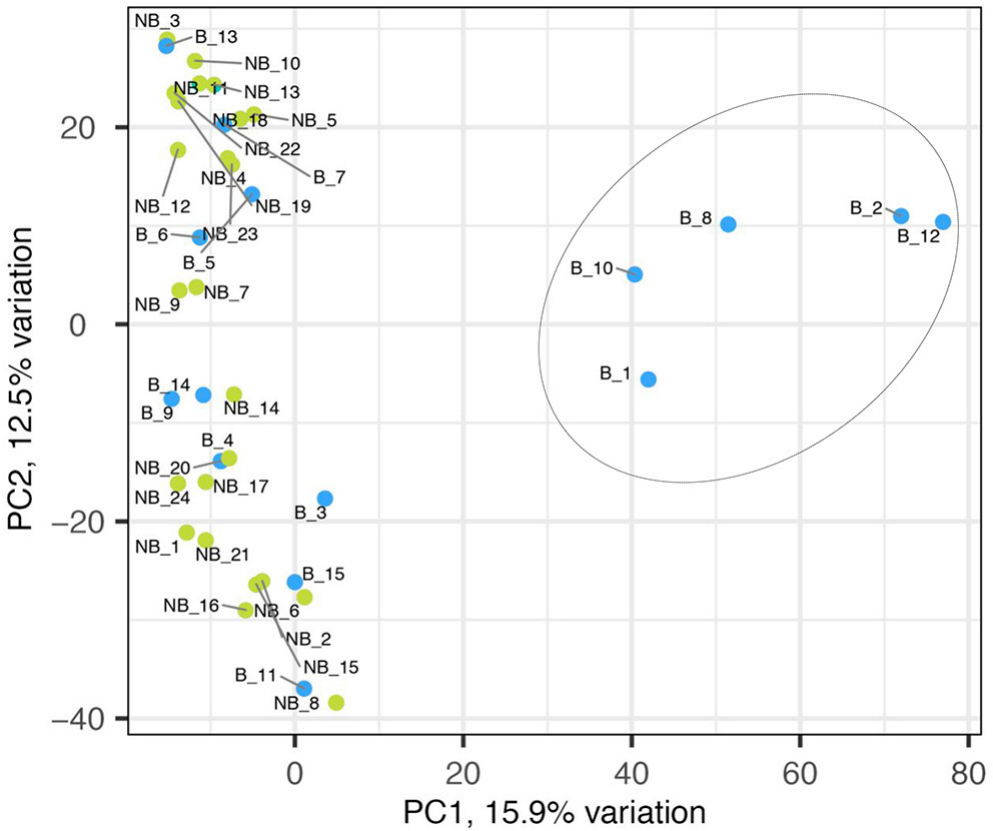
Principal component analyses of 10,924 SNP markers on bolter and non-bolter phenotypes of L14 plants. Each dot represents a sample, and the blue color represents bolters (B) while the green color represents non-bolters (NB). The first two axes of the PCA explain 28% of the overall variance and separated the bolter and non-bolter phenotypes.

The sequencing of 15 bolting and 24 non-bolting individuals of the L14 line, identified as the best material from phenotyping, generated a total 10,924 SNPs based on the EL10 reference genome (NCBI PRJNA413079). The SNP markers had a high call rate of 97% across all the samples and were 100% reproducible *in-silico*. A crude usage of the different alleles of the 10,924 SNP markers to understand the genetic structure resulted in the separation of bolters and non-bolters of L14 (Figure 2). The first two axes of the PCA contributed to 28% of the overall variance. This allowed us to proceed with further analysis to determine the strength of association of SNPs with the trait of lower bolting tendency.

### GWAS for the Identification of SNPs Linked to Post-winter Bolting Tolerance

**Figure 3:**
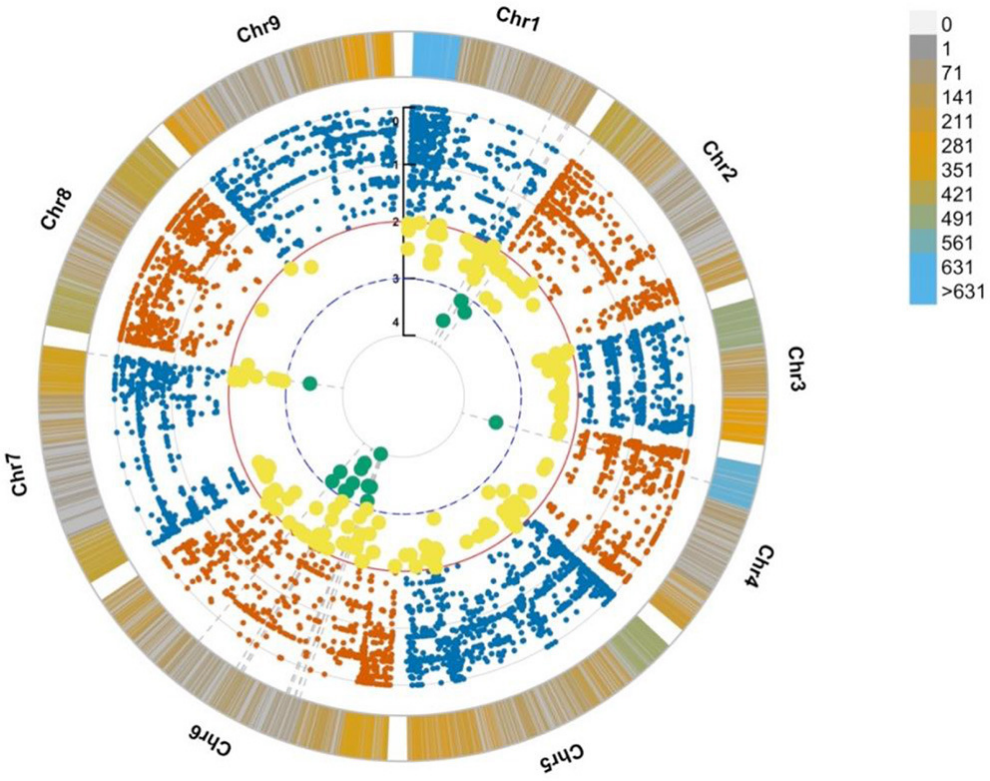
Circular Manhattan plot from association analyses showing SNP density per chromosome along with significant SNP associations. The bars along the chromosomes present the SNP density in a window size of 10 M base pairs. The most significantly associated SNPs are highlighted in yellow (*p* ≤ 0.01) and cyan (*p* ≤ 0.001).

The genotypes of all the identified SNPs were used to build an association analysis resulting in the identification of key SNPs discriminating the bolting from the non-bolting individuals of the L14 line. The assumption was that the most significant SNPs, located in key causal genes, should ideally show a discrete separation of genotype calls or patterns of association. The model also considered rare alleles (MAF < 0.05). These variants were mined using two likelihood ratio tests: (i) Fisher exact test and (ii) nbinom test (Supplementary Figure 1). The results from the association analyses indicated that the SNP density was found to be the highest at the beginning of chromosomes 1 and 4, and the most significant SNP associations based on differential allele frequencies were found in chromosomes 2, 4, 6, and 7 (Figure 3, cyan dots). This was consistent with the identification of markers associated with flowering time and bolting phenotype reported by other authors (Abou-Elwafa et al., 2012; Pfeiffer et al., 2014; Tränkner et al., 2017). Further, the annotations of the genes harboring the SNP markers based on the EL10 genome resulted in the implication of flowering-related genes and loci. More specifically, seven genes were involved in vernalization responsiveness, six genes were related to flower development, and 13 other genes were found involved in flowering time. These observations have been summarized in Supplementary Table 2. For downstream validation based on molecular methods, common SNPs resulting from the two statistical methods were considered and further filtered based on their degree of association using the *p*-value (Supplementary Figure 1).

### Molecular Validation of Identified Single Nucleotide Polymorphisms Using Different Single Nucleotide Polymorphism-Based Technologies

The molecular validation strategy involved three technologies: (1) high-resolution melting analysis (HRM), (2) Sanger sequencing, and (3) SNP genotyping using rhAmp based assays.

**Figure 4:**
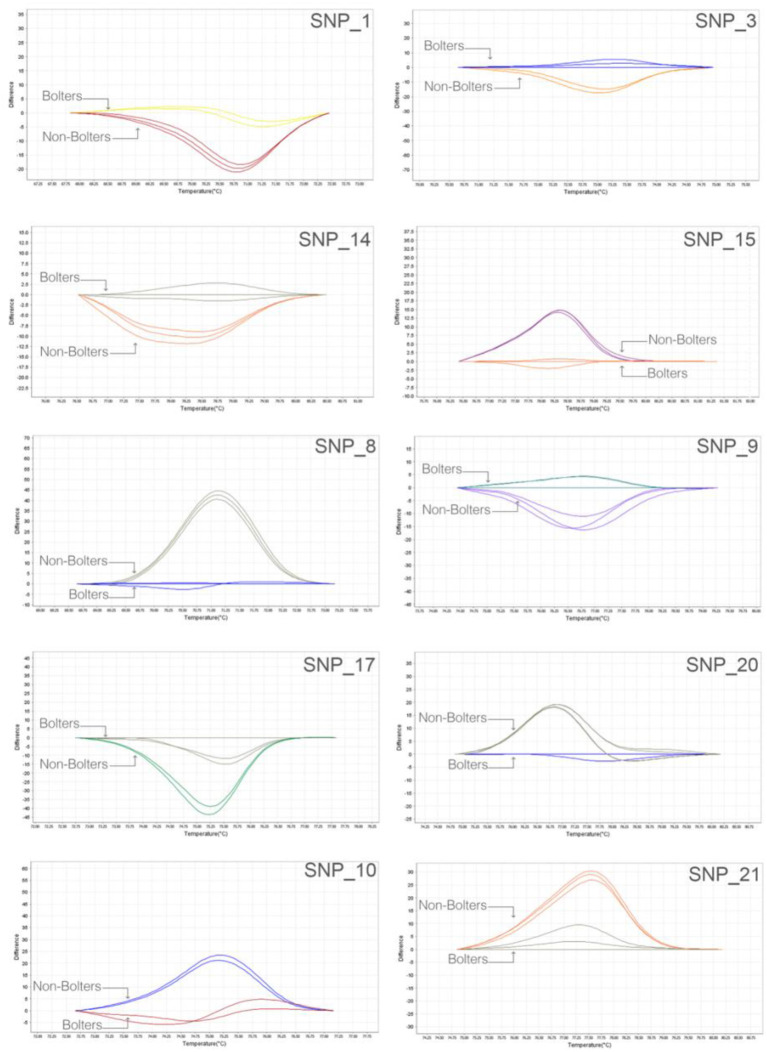
Robust and quick SNP screening by high-resolution melting (HRM) of selected SNP targets: representative melt curve profiles discriminating three biological replicates of bolters and non-bolters from the L14 line are presented here as an example. The two different melt curve colors for each SNP target correspond to samples of bolters and non-bolters labeled respectively.

We adopted a rapid and reliable approach to screening SNP markers identified from the bioinformatic association analyses using, as the first step, 384-well plate based high-resolution melting (HRM) assays. Thirty SNP targets were evaluated on nine individuals of bolters and non-bolters of the L14 line in triplicates. The difference in the melt curve profiles of the bolter and non-bolter phenotypes of L14 as a result of their sequence composition can be seen in Figure 4 for some of the best discriminating SNPs. The largest differences in the melting temperatures between the bolters and the non-bolters were weighed using by non-parametric test (*T*-test; Supplementary Figure 2) for the discriminating SNPs to proceed with Sanger sequencing-based validation.

**Figure 5:**
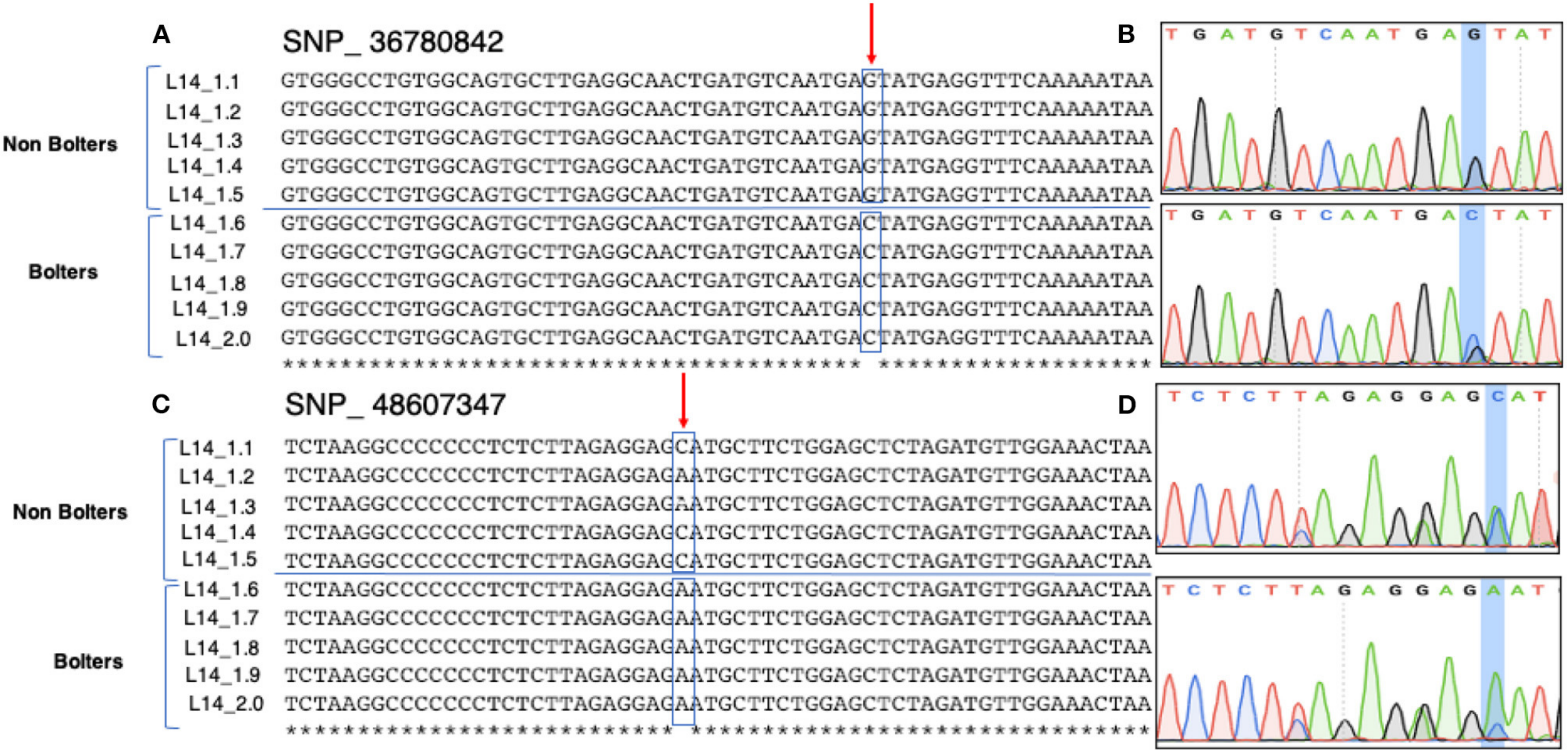
Sanger sequencing-based validation of SNP_36780842 (Chr1, position 36780842) and SNP_ 48607347 (Chr2, 48607347) on five biological replicates of bolters and non-bolters of the L14 line. Top: **(A)** multiple sequence alignments and **(B)** the corresponding chromatogram of SNP_36780842 show non-bolters of the L14 line with the G allele. Bottom: **(C)** multiple sequence alignments and **(D)** corresponding chromatogram of SNP_48607347 show non-bolters of the L14 line with the C allele. Both SNPs were found to be segregating within the L14 line.

Sanger sequencing-based validation was carried out for 10 promising SNP targets from HRM based validation (Figure 4). Figure 5 shows the multiple sequence alignments and representative chromatograms of two SNPs, SNP_36780842 (top) and SNP_48607347 (bottom), which were best substantiated between the bolters and the non-bolters of L14. For SNP_36780842, the non-bolters of the L14 line showed the G allele, while the bolters showed the C allele (Figure 5A), which is also confirmed in the chromatogram (Figure 5B). Concerning SNP_48607347, most of the non-bolters presented the C allele, while all the bolters showed the A allele (Figure 5C), a representative chromatogram is also shown (Figure 5D).

To further understand the association of these two SNPs with bolting tolerance, we developed genotyping assays corresponding to the SNPs validated by Sanger sequencing. The validation was performed on three sets of plant material to understand their ability to discriminate (1) annual vs. biennial habits, (2) bolters- vs. non-bolters of biennial habits, and (3) spring-sown and autumn-sown hybrid varieties. The results of the association test with chi-square and p-value are reported in Table 2. Both the SNPs described in this study, SNP_36780842 and SNP_ 48607347, were found to be significantly associated with the three validation datasets tested for bolting tendency. The allele frequencies observed were highly statistically significant between annual and biennial beet individuals, bolter and non-bolter beet individuals, and for spring-sown and autumn-sown varieties (*p* < 0.0001) as shown by the contingency table (Table 2).

**Table 2:** Contingency table based on rhAmp genotyping of SNP_36780842 and SNP_48607347 at three levels—annual vs. biennial beets, bolters and non-bolters of the biennial beets, and autumn-sown and spring-sown varieties.

**SNP_ 36780842**		**C**	**G**	***Chi-square***	***p-value***
Validation dataset 1	Annual	**83**	**9**	***43.34***	*** <0.0001***
	(*n* = 46)	**90.20%**	**9.80%**		
	Biennial	**40**	**52**		
	(*n* = 46)	**43.50%**	**56.50%**		
Validation dataset 2	Bolters	**196**	**30**	***80.95***	*** <0.0001***
	(*n* = 113)	**86.73%**	**13.27%**		
	Non-bolters	**84**	**102**		
	(*n* = 93)	**45.16%**	**54.84%**		
Validation dataset 3	Autumn-sown	**268**	**116**	***126.34***	*** <0.0001***
	(*n* = 191)	**70.15%**	**30.00%**		
	Spring-sown	**379**	**3**		
	(*n* = 192)	**98.69%**	**0.78%**		
**SNP_ 48607347**		**C**	**A**	***Chi-square***	***p-value***
Validation dataset 1	Annual	**12**	**78**	***94.74***	*** <0.0001***
	(*n* = 46)	**13%**	**85%**		
	Biennial	**80**	**14**		
	(*n* = 46)	**87%**	**15%**		
Validation dataset 2	Bolters	**81**	**107**	***25.25***	*** <0.0001***
	(*n* = 94)	**43%**	**57%**		
	Non-bolters	**148**	**70**		
	(*n* = 108)	**69%**	**32%**		
Validation dataset 3	Autumn-sown	**234**	**144**	***29.66***	*** <0.0001***
	(*n* = 189)	**61.90%**	**38.09%**		
	Spring-sown	**162**	**222**		
	(*n* = 192)	**42.18%**	**57.81%**		

*The bold values represent the count of individuals obtained in each category along with a percentage calculated based on the total number of individuals test. The chi-square value provides a measure of the correlation between the categorial variables (the phenotype and the allele of the SNP in each case), and the p-value indicates if the statistical test resulted significant (p < 0.05)*.

**Figure 6:**
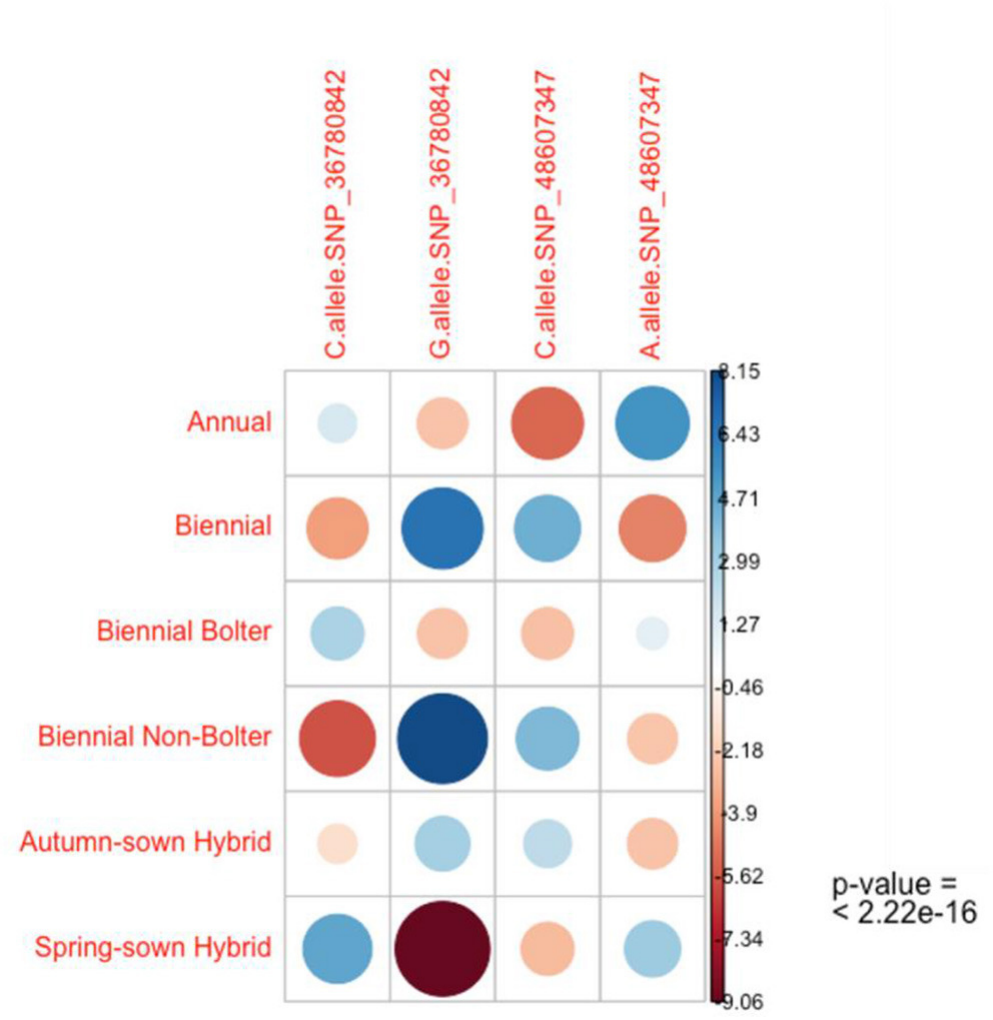
The chi-square test of independence reveals the nature of dependence of the alleles with the phenotypic categories of sugar beets. A strong positive association of the G allele of SNP_36780842 is seen in the biennial, biennial-non-bolter, and autumn-sown categories contrary to the strong negative association seen in the spring-sown varieties and moderate negative association observed in biennial bolters and annual beets. Similarly, a moderate positive association of the C allele of SNP_48607347 is seen in the biennial, biennial-non-bolter, and autumn-sown categories inverse to the negative association seen in the spring-sown variety, biennial bolter and annual individuals. The *p*-value of the standardized residuals is significant (*p* < 0.001).

The chi-square test of independence was used to analyze the contingency table formed by the evaluated alleles and phenotypes as categorical variables. Particularly, this test evaluated whether there is a significant association between the categories. As the output of this test, the mosaic plot in Figure 6 shows the combination of positive and negative associations between phenotypes, alleles C and G of SNP_36780842, and alleles C and A of SNP_48607347. It is interesting to observe the strength of positive association of the low bolting allele G of SNP_36780842 in biennial, biennial non-bolter, and autumn-sown categories. The exact opposite of a strong negative association is seen between C of SNP_36780842 and spring-sown varieties, biennial bolters, and annual beets. With respect to SNP_48607347, the contribution of the C allele to the biennial, biennial non-bolter and autumn-sown hybrid categories can be well-appreciated differing from the negative correlation from the A allele observed in annual beets, biennial bolters, and spring-sown varieties (Figure 6). A qualitative separation of the alleles for the bolter and non-bolter checks is also shown in Supplementary Figure 4. These associations further confirmed observations from Sanger sequencing (Figure 5). Bolting tolerance in biennial beets can, thus, be linked to a higher frequency of the G allele at SNP_36780842 and a higher frequency of the C allele for SNP_48607347.

**Figure 7:**
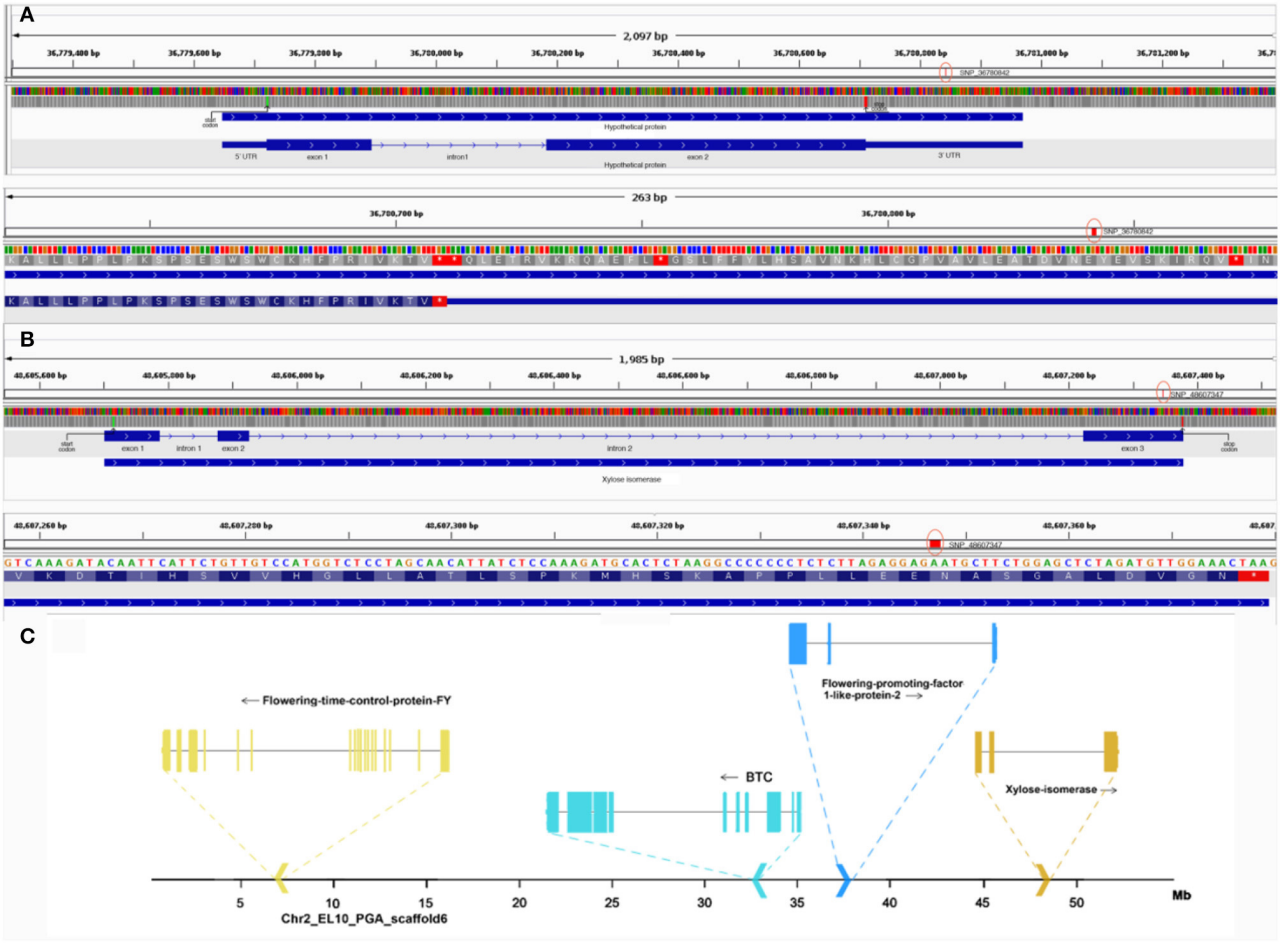
**(A)** Top: gene structure of hypothetical protein in Chr1 containing SNP_36780842. Here, the green line represents the start codon, red line represents the stop codon and red circle highlights the SNP. Bottom: closer view of SNP_36780842 in context of the 3' UTR. **(B)** Top: gene structure of xylose isomerase in Chr2 containing SNP_48607347. Here, the green line represents the start codon, the red line represents the stop codon, and the red circle highlights the SNP. Bottom: closer view of SNP_48607347 in exon 3 of the gene. **(C)** Chromosomal context of other flowering-related genes in chromosome 2 near the locus of xylose isomerase containing SNP_48607347 is highlighted.

The strong association of the two SNPs markers compelled the authors to also investigate the molecular effect of the SNP and understand its chromosomal context. SNP_36780842, located within a gene coding for a hypothetical protein in chromosome 1, was found to be a 3'UTR variant (Figure 7). To annotate the gene, an ortholog search in *Arabidopsis* was done and pointed to AT1G47410, which was still uncharacterized in terms of function and protein domains. In fact, orthologs in many species remained uncharacterized but provided a 75% overlap between the protein in question and Chaperone J-domain superfamily proteins. Further, PSI-BLAST resulted in its annotation as “DNAJ heat shock N-terminal domain-containing protein” in *Prunus dulcis*. The protein similarity and overlaps along with the ortholog tree specifically looking at orthologs of *B. vulgaris, Arabidopsis*, and *Prunus* can be observed in Supplementary Figure 3. The second validated SNP, SNP_48607347, located in chromosome 2 of sugar beet was found to be a non-synonymous variant in exon 3 of the gene encoding xylose isomerase changing the amino acid arginine to histidine (Figure 7). Non-synonymous variants more often end up having functional consequences ranging from protein structure to enzyme activity because of amino acid substitution. The gene xylose isomerase is located ~10 Mb from other important flowering-related genes in sugar beet (Figure 7), such as *BvBTC, BvFT1*, and *BvFT2* (Pin et al., 2012; Dally et al., 2018; Höft et al., 2018b).

## Discussion

In *B. vulgaris* L., there is a trade-off between sugar yield and bolting, which is the first visible sign of reproductive transition (Mutasa-Göttgens et al., 2010). Specifically, the cultivation of sugar beet as a winter crop is considered far-fetched, primarily because of bolting. Further, large variations have been found in the natural beet populations consisting of a mixture of semel- and iteroparous plants with different reproductive behaviors (Hautekèete et al., 2002; Van Dijk, 2009; Dijk and Hautekèete, 2014). These culminate in the complex genetic and epigenetic control of bolting, vernalization and flowering time in sugar beet. While the candidate genes for these processes are well-mapped in the model species *Arabidopsis thaliana*, it is still being investigated in an important crop like sugar beet (Pfeiffer et al., 2014; Höft et al., 2018a).

The goal of this study was to identify candidate genes and validate SNP alleles linked to low bolting tendency after autumnal sowing and vernalization. The lack of prior information on bolting-tolerant sugar beets post-winter urged the authors to compare available sugar beet materials for their survival rate and bolting percentage post-winter. Thus, a 3-year field trial was organized and resulted in the identification of a suitable plant material, L14, to further understand the molecular basis of tolerance to bolting post vernalization.

The use of contrasting phenotypes of the L14 line and high-throughput sequencing resulted in the identification of a large number of SNPs (10,924). Mining the most useful SNPs discriminating the phenotypes involved the use of both (a) Fisher exact test and (b) nbinom test from DESeq2 to identify SNP signatures strongly linked to bolting tolerance post-winter (Supplementary Figure 1). Several other studies using similar test statistics resulted in the identification of good causal markers (Liang et al., 2016; Yang et al., 2016; Lee et al., 2018).

While we confirmed the strong association of two markers in this study on three validation datasets containing contrasting bolting phenotypes, we were also able to identify and map candidate genes related to vernalization responsiveness, flowering time, and floral development through bioinformatics analyses (Supplementary Table 2). SNP_36780842 was found to be located within the 3' UTR of a gene coding for a hypothetical protein in chromosome 1 (Figure 7). The importance of the 3' end of genes in transcription control and protein targeting is well-understood in model organisms. Particularly, the different modes of 3' RNA processing and flowering time control were reviewed with respect to the FLC in *Arabidopsis* (Rataj and Simpson, 2014). Post-transcriptional coordination in the control of flowering time through 3' UTR-mediated decay of flowering time control protein *SOC1* was shown in another study (Kim et al., 2013). However, since the gene under study was uncharacterized for its function and domains by most preliminary search tools and bioinformatics prediction of orthologs for the same resulted in 75% overlap with proteins constituting the chaperone-J-domain superfamily (Supplementary Figure 3). The role of J-domain-containing proteins and their relevance in flowering have been shown in many studies (Yang et al., 2010; Shen et al., 2011; Pulido and Leister, 2018).

It was equally interesting to understand the involvement of SNP_48607347 in flowering (Figure 7), located within the coding region, exon 3 of xylose-isomerase gene in chromosome 2 of sugar beet. This enzyme is important for the utilization of sugar substrates as shown in *Arabidopsis thaliana* (Cho et al., 2018). We identified a non-synonymous mutation in the xylose isomerase gene possibly affecting the protein structure because of the substitution of arginine with histidine (Figures 5, 7). It has also been previously shown that mutations in certain starch metabolic genes may result in late flowering (Yu et al., 2000). To further understand its biological relevance, we looked at literature related to the effects of point mutations in xylose-isomerases. In a patent application (US20160040151A1), point mutations resulting in the substitution of asparagine to histidine were shown to increase xylose utilization in yeast. More evidence supporting a tight link between sugar metabolism and late-flowering has been reported in a recent study (Seo et al., 2011). In this study, it was shown that sufficiently high levels of sugar resulted in flowering in *Arabidopsis*, and a drop in sugar levels beyond a threshold resulted in prolonged vegetative growth. Taken together, it could be reasoned that non-bolters presenting the amino acid histidine might be better utilizers of sugar resulting in prolonged vegetative growth. Since xylose is the second most abundant sugar source in plants, we hypothesize the involvement of xylose-isomerases in utilizing available sugars, such as arabinose and xylose, to modulate endogenous sugar levels and metabolism important in the complex signaling for floral transition.

In addition to understanding the molecular effect of the validated SNPs, we have provided the flanking sequences of all significantly associated SNPs for use as a panel associated with low bolting tendency in Supplementary Text 1. The availability of SNPs from this study linked to bolting resistance provides further insights into bolting tolerance, vernalization responsiveness, and flowering in sugar beet. They can have a compelling impact on the breeding process, considering that the selection of high yielding sugar beet varieties against bolting is highly needed. This becomes even more necessary when many genotypes are to be tested or in the case of genetic purity assessments in seed companies.

To the knowledge of the authors, this study led to the identification of (1) sugar beet lines with high survival rate and bolting tolerance post-winter, (2) SNP alleles associated with low bolting tendency post-winter, and (3) description of candidate genes involved in bolting resistance in sugar beet.

## Materials and Methods

### Plant Materials and Experimental Field

The plant material used in the phenotyping for bolting tolerance after autumnal sowing from a three-year field trial consists of 13 sugar beet lines provided by the Department of Agronomy, Food, Natural Resources, Animals and Environment, University of Padova, Italy and is summarized in Supplementary Table 1. All lines were pollinators, diploid, and endowed with resistance to rhizomania. There are pollinators carrying the allele for biennial habit at the *BTC1* locus in the homozygous state (Stevanato and Biscarini, 2016). One line selected from phenotyping was then involved in the RAD-sequencing and association analysis aimed to identify SNPs associated. For validating the identified SNP markers, the plant material comprised seven annual sea beets and 14 biennial sugar beet varieties further consisting of both bolting and non-bolting individuals. The seven annual *B. vulgaris* subsp. *maritima* populations were sampled along the Adriatic Sea coastline of Italy and Croatia. The sampling of populations was performed according to Bartsch et al. (1999). These populations, separated by at least 15 km or by physical barriers, were considered distinct. The seeds were collected from randomly selected plants more than 5 m apart for each population. The seeds were cleaned and stored at 7°C and 30% relative humidity. These populations are carrying the allele for annual habit at the *BTC1* locus in the homozygous state (Stevanato and Biscarini, 2016). The annual and biennial material used for validation is presented in Supplementary Table 5.

The experimental site for the 3-year field trial was located in Minerbio, Bologna, Italy (N44.629902, E11.552059). The seeds of each genotype were sown on November 19–20, 2016 for the 1st year, November 20, 2017 for the 2nd year, and November 22, 2018 for the consecutive year. Sugar beet plants were thinned after germination in the field to obtain 250 individuals per line distributed in three randomized plots. The plots were managed following local recommendations until the bolters and survival count date. The final count was carried out in the middle of June across the 3 years. Plants displaying stem elongation were scored as bolting individuals, while plants that did not show stem elongation were labeled as non-bolting individuals (Table 1).

### Genotyping by Sequencing of L14 Bolters and Non-bolters

Genomic DNA was obtained from 15 bolting and 24 non-bolting individuals of the L14 line using a modified CTAB DNA extraction method (Doyle and Doyle, 1990). The quality of the extracted DNA was checked on a 0.8% agarose gel, and quantification of the same was done using a Qubit 4.0 fluorimeter (Thermo Fisher Scientific, Waltham, MA, Unites States). The DNA samples were subjected to a Restriction site-associated DNA technology (RAD-seq) with a HiSeq 2000 sequencing system using 150-bp paired-end sequencing (Illumina Inc., San Diego, CA, United States). Briefly, the extracted genomic DNA was digested using PstI (New England Biolabs, Ipswich, MA, United States) and ligated with Illumina RE-site compatible P1 adapters (Illumina Inc., San Diego, CA, United States). The size selection of 300–500 bp was performed using solid-phase reversible immobilization (SPRI) beads (AgencourtAMPure XP Beads) from Beckman Coulter (Indianapolis, IN, United States). The resulting fragments were end-repaired, 3′-adenylated, and ligated with universal P2 adapters. The libraries were diluted and pooled with an equimolar concentration of each library and sequenced on an Illumina HiSeq 2500 platform (Illumina, San Diego, CA, United States) following the recommendations of the manufacturer.

### Bioinformatics Data Analyses

Variant calling was performed using an in-house pipeline, which uses fastqc (version 0.11.5), bowtie2 (version 2.3.5.1), samtools (version 1.9), picard-tools (version 2.18.12), samtools (version 1.9) mpileup, and bcftools (version 1.9) to extract variants. The resulting vcf files were filtered using bcftools (version 1.9). The reference genome used was the EL10 Sugar Beet reference genome (NCBI PRJNA413079). The base file for all the bioinformatics analyses described was the merged vcf file containing genotypes of the 10,924 markers across 15 bolters and 24 non-bolters. The genotype matrix was analyzed using the SNPRelate package in R to build PCA plots and understand the genetic structure (Figure 2). For the genome-wide association analyses, the genotypes for each of the SNP markers were reduced to allele frequencies using the genetics package in R. Statistical Fisher test of independence using custom R scripts (attached as Supplementary Text 2) was then conducted to test the difference of partial association of genotypes in two strata of contingency tables, corresponding to two groups of bolters and non-bolters (Liang et al., 2016; Yang et al., 2016; Lee et al., 2018). Manhattan plots to visualize the data were generated using the qqman package in R (Figure 3). Then, to come up with the best SNP signatures discriminating against the two phenotypes, the results from the SNP-trait association analyses were also processed with the DESeq package using the pairwise nbinom function. A subset of highly associated SNPs (*p* < 0.01) was selected as candidates for downstream molecular validation (Supplementary Text 1).

The commonly resulting signatures from the association study and DESeq approach (Supplementary Figure 1) were also used in SNP effect prediction and gene annotation to catalog genes related to flowering in sugar beet (Supplementary Table 2). With respect to the annotation of orthologs of genes identified here, OrthoDB (v9), TAIR-BLAST (2.9.0 +), and PSI-BLAST (hosted at NCBI) were used to compile the details presented in Supplementary Table 2. For SNP effect prediction, the snpEff tool (version 5.0) was used, and effects were predicted using the custom-built EL10 reference genome and gff file. Flanking sequences of significantly associated SNPs are shared in Supplementary Text 1. Visualization of the gene structures and SNPs was done using IGV (version 2.9).

## Molecular Validation

### High-Resolution Melting Analysis

Flanking sequences of 150 bp around the target SNP were extracted using the bedtools getfasta tool (version 2.28.0). The list of sequences was given as input to the stand-alone version of the Primer3 software (version 4.0) to design primers with suitable amplicon lengths. The HRM primer sequences are available in Supplementary Table 3. Nine biological replicates of biennial bolters and non-bolters of the L14 line were tested in triplicates. The HRM analyses were carried out in 384 well plates on the QuantStudio 12K Flex (Life Technologies, Carlsbad, CA, United States). Reactions were carried out using a 5 μL reaction volume comprising 2.5 μL of MeltDoctor™ HRM Master Mix (Thermo Fisher Scientific, Waltham, MA, United States), 0.45 μL of 10 μM forward and reverse primer, and 1.6 μL of sample DNA of concentration 5 ng/μL. The thermocycler program consisted of 10 min of pre-incubation at 95°C, followed by 40 cycles of 15 s at 95°C, and 1 min at 60°C. This was followed by the melt curve of 10 s at 95°C, 1 min at 60°C, 15 s at 95 °C, and 15 s at 60°C. The melt curve profiles were analyzed using the HRM software. The primers showing a significant difference in Tm between bolters and non-bolters, established by a non-parametric statistical test (*T*-test; Supplementary Figure 2), were taken downstream for validation based on Sanger sequencing, and their sequences are available in Supplementary Table 4.

### Sanger Sequencing

Flanking sequences of 250 bp around the target SNP were extracted using the bedtools getfasta tool (version 2.28.0). The list of sequences was given as input to the stand-alone version of the Primer3 software (version 4.0) to design primers with suitable amplicon lengths. Five biological replicates of bolters and non-bolters of the L14 biennial line were tested. The reaction setup used comprised of 10 μL of 5X Reaction Buffer (PCR Biosystems Ltd., London, UK), 2 μL each of 10 μM forward and reverse primers, 2 μL of 2u/μl of HiFi Polymerase (PCR Biosystems), 5 μL of 50 ng/μL sample DNA made up to a 50 μL using nuclease-free water. The thermocycler program consisted of 1 min of initial denaturation at 95°C, followed by 30 cycles of 15 s at 95°C, 30 s at 60°C, and 30 s at 72°C. The amplicons were run on a 1.5% agarose gel to verify the amplification. PCR products were purified using NucleoSpin Gel and PCR Clean-up (Macherey-Nagel, Bethlehem, PA, United States) and were sent for sequencing. The resulting sequences were analyzed using ClustalW2 (http://www.ebi.ac.uk/Tools/msa/clustalw2/) and SnapGene 5.1.7 (Chicago, IL, United States) to generate multiple sequence alignments and chromatograms (Figure 5).

### Genotyping

Validated SNPs from Sanger sequencing were used to design rhAmp assays (Integrated DNA Technologies, United States). Their sequences are available upon request. Three hundred biological samples were subjected to rhAmp genotyping, which was performed in 5 μL using 384-well plates, and low Rox was used as a passive reference dye. 5 ng of DNA was mixed with 2.65 μL of rhAmp Genotyping Master Mix, 0.25 μL of rhAmp SNP assay mix, and 1 μL of nuclease-free water. The thermal cycle parameters were adopted from Broccanello et al. (2018). The association between the two SNPs and phenotypes of bolting was also evaluated and confirmed by chi-square statistics using the chi.square() function in R. Chi-square residual analyses and visualizations were performed using the library corrplot and gplots.

## Data Availability Statement

The datasets presented in this study can be found in online repositories. The names of the repository/repositories and accession number(s) can be found at: https://www.ebi.ac.uk/ena/browser/view/PRJEB42403.

## Author Contributions

SR, GCa, and PS made the experimental design. SR, GCa, MD, GB, and PS carried out the sampling. SR, CB, CC, LM, AE, and MM performed molecular analyses. SR conducted the statistical and bioinformatics analyses and wrote the study. AS, GCo, SS, and PS contributed to the critical writing and review of the manuscript. All the authors reviewed the manuscript and gave final approval for publication.

## Conflict of Interest

The authors declare that the research was conducted in the absence of any commercial or financial relationships that could be construed as a potential conflict of interest.
