# Supplementary material for: SNP Alleles Associated With Low Bolting Tendency in Sugar Beet
Source: Front Plant Sci. 2021 Jul 12;12:693285. doi: 10.3389/fpls.2021.693285 (PMC8311237; doi:10.3389/fpls.2021.693285)
Supplement: Supplementary file 1 [file Data_Sheet_1.docx]

**Supplementary Figure 1: Common SNP signatures found between two bioinformatics pipelines to strengthen the identification of markers related to low bolting tendency. Pipeline 1 used nbiom test from DESeq2 on the genotype matrix to identify significant SNP signatures while Pipeline 2 used Fisher test on the genotype matrix to determine significant SNPs**


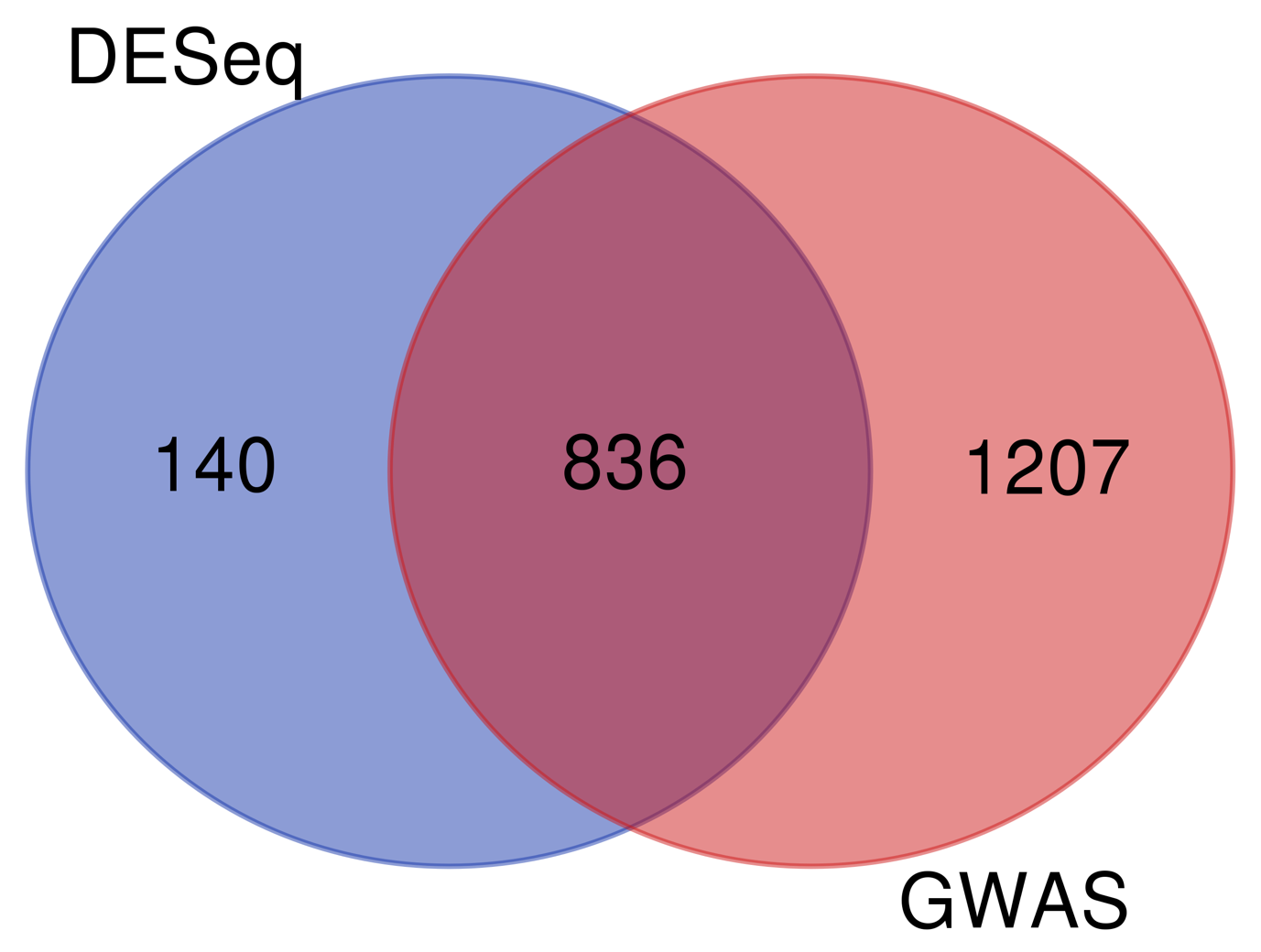


Pipeline 2

Pipeline 1

**Supplementary Figure 2: Bar plots of discriminating SNPs between bolters and non-bolters upon HRM detection for downstream validation based on Sanger sequencing**

**
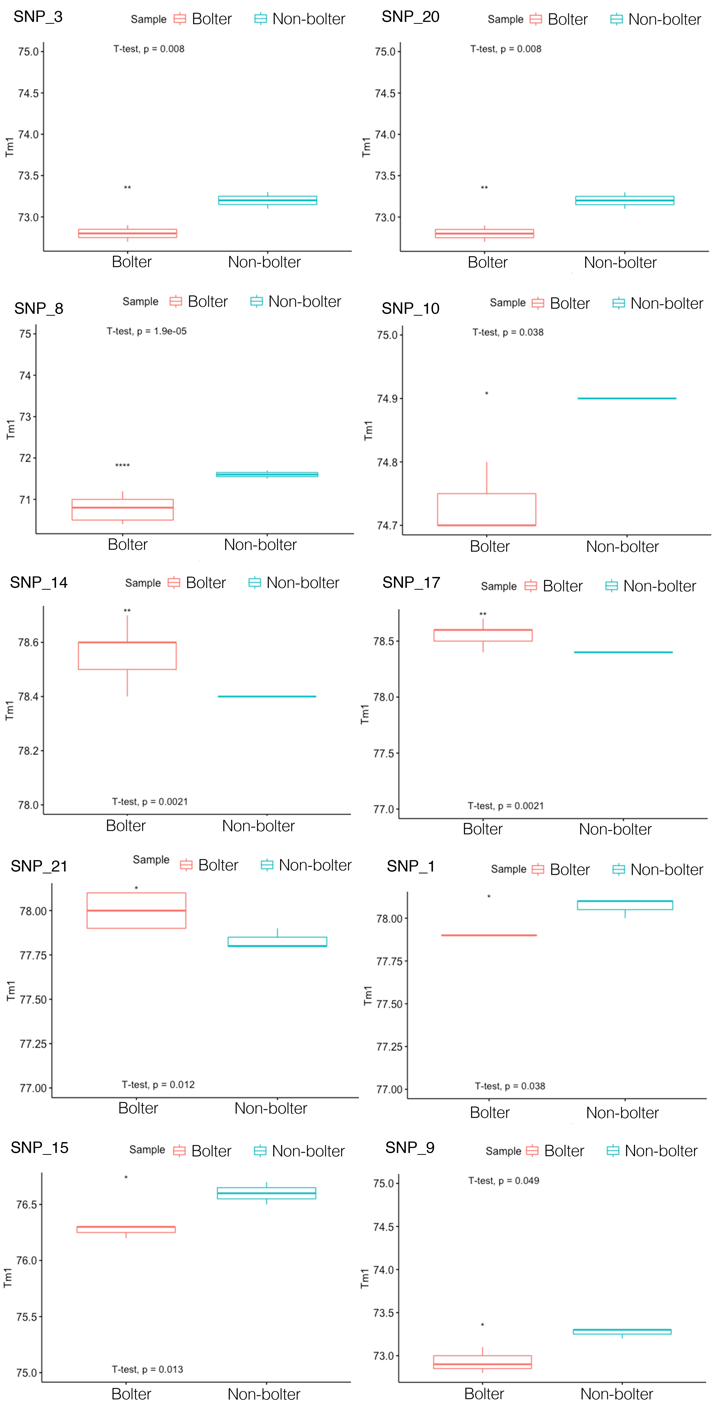
**

**Supplementary Figure 3: A) PSI-BLAST hits for the hypothetical protein of *Beta vulgaris* B) Gene tree of the orthologs based on hypothetical protein of *Beta vulgaris showing the* nearest annotated ortholog as DNAJ domain containing heat shock protein *in Prunus dulcis* along the protein sequence alignment seen conserved across the different species**

**A)**

***
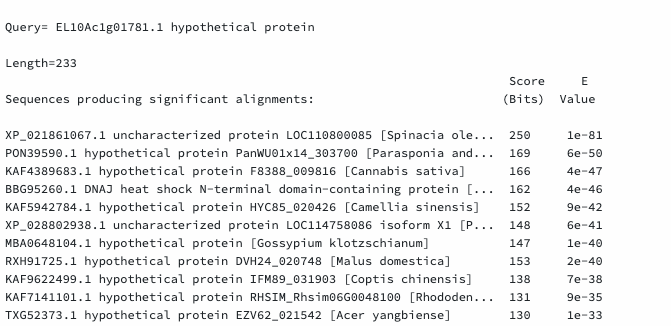
***

**B)**

**
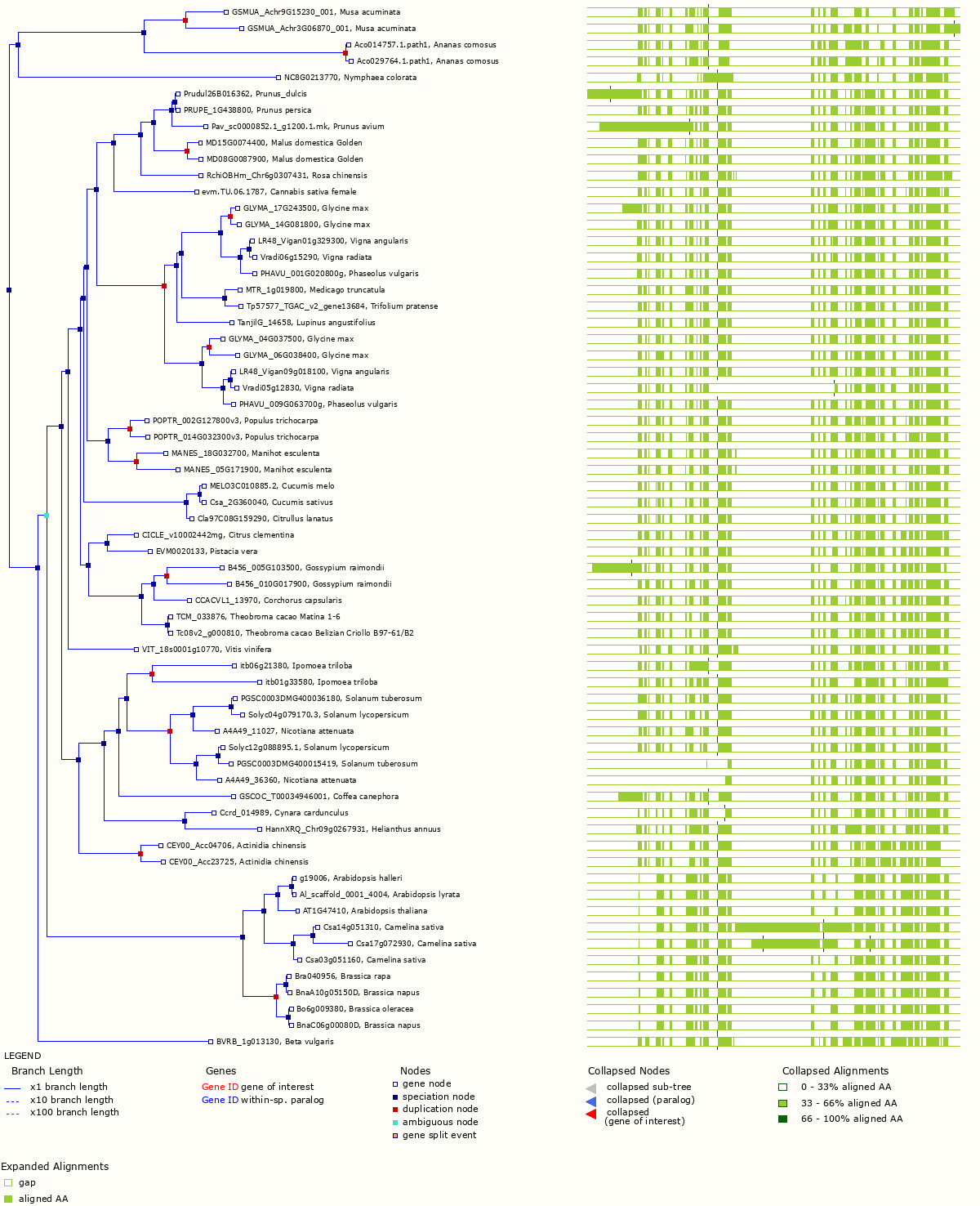
**

**Supplementary Figure 4: Qualitative representation of allelic discrimination assays of A) SNP_36780842 and B) SNP_48607347 on representative varieties classified as bolters and non-bolters**

**
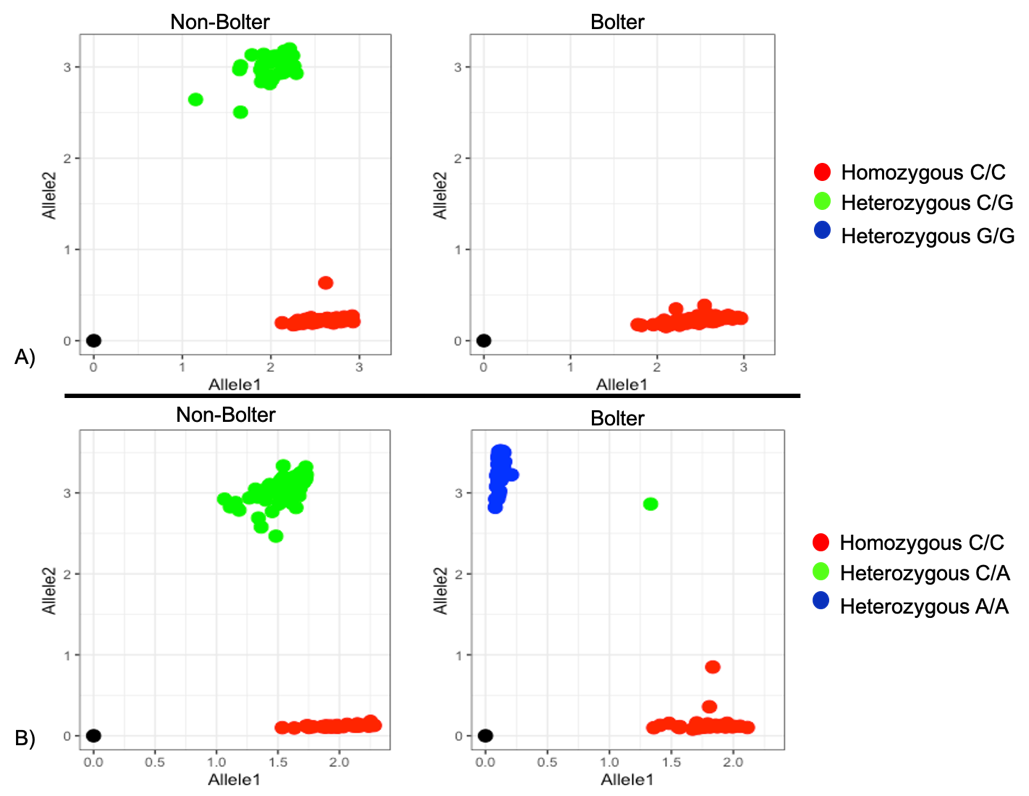
**

**Supplementary Text 1: Sequences of flanking regions of significantly associated SNPs obtained from association analyses**

>SNP31

TGCAGAAGATTTTGAAAGAATCTTCTACTT[A/T]TTAGTTTCCCAGGATATCGAAAAAGGCCTGAGAAGACA

>SNP33

TGCAGAAGGATTATCAAGTTTCCATCAAAGATTTTACTGCTTTCATAAAT[C/A]GATTGTGCAAGAGAGGTT

>SNP34

TGCAGATCAGCAAAAATTGTCAAG[C/G]ATGTGGAACTACTTCTTCTACCCTGTTTTCCATTGGATACAATT

>SNP32

TGCAGATCTGAAAAAATTGCAAATATGTTGC[A/G]TCACTATGTCCTCAGTCCCCACCTTCATGGGCTGTAC

>SNP35

TGCAGATGATCCAAATACTC[C/A]CCTTCCAGAGCATCGAAAGAATTGTAGACGTATCCCCAACTCTTGGAA

>SNP2

TGCAGCCTAGTATCTAAAAGAAAGGATGGTG[G/A]CTATCAAGAAGCATACACACTTGGGGAAAGAGAATGT

>SNP36

TGCAGCCTTCTCTTCACAGTGTGAACGTTATCACTTCGATC[C/T]AGCTCCATTCCCAACACACAGCCAGTA

>SNP37

TGCAGGAAAAGACCTCATGTCAGAAACACACCTTAGCAACCTAAGATCTGT[C/T]TGTCCAGCAGTTGGGGG

>SNP38

TGCAGGCAGCAATGCCTTTGTGATGGGGAATCCTTC[T/C]AAGCCCCAGAAATGGCATGTTTACTGTGCATC

>SNP39

TGCAGTTATAAACTTTGACTTG[C/A]TATGAATAGGAATGGATGATGGGTGTTATGACATTATGTGAATTTC

>SNP40

TGCAGAAAAAGGTATTGG[G/A]TCAAGGACGTGTATACATCATCAGACCATTACAGATCGGAAGAGCGGTTC

>SNP41

TGCAGGACATATTGCAAATTGGTTCTGGAGTATATAA[T/G]TTCATGGGAAACTGTTACAGATCGGAAGAGC

>SNP42

TGCAGACTTAGGATCTTCACCATCTTCGATCCCCCCCTAATTATAGTCGTATAACAAGTGATAAGCA[G/T]A

>SNP43

TGCAGCAAG[T/C]ACTTTGGAAATTAGGAAGGAAGAAAACAAAGAAAAGTAACAGAAACATTGCCTGCATGA

>SNP44

TGCAGGTAATCAAACAGTCCAAAAAGTTCTGT[T/C]CTGAAGCTTTGAAATTTCTCAAGGCTTTACTTCTTG

>SNP45

TGCAGGTTCTAGTCTCAATGGTAACCTTGTTAGTTTGTATTTCCTTTCAAATTTCA[A/G]TAAAGTTCTTTT

>SNP46

TGCAGTTCAATGTCCTATTGTCTTTCACTTTGCTAAACTTCCTCTAATTGGAAATAGG[C/T]ATGTATCACT

>SNP47

TGCAGTTCAATGTCCTATTGTCTTTCACTTTGCTAAACTTCCTCTAATTGGAAATAGGCA[C/T]GTATCACT

>SNP48

TGCAGTAAT[C/T]CATACTTCATTTTGGCCCAAACAAAAATCATGTCGCCACTAGGACAAAAGCTGTTTTGT

>SNP49

TGCAGTAATTCATACTTCATTTTGGCCCAAACAAAAATCA[T/C]GTCGCCACGAGGACAAAAGCTGTTTTGT

>SNP_50

TGCAGTTTAGAATTTTACAATTTTTTTGTTGTATGTGGAAGTAGCTCATTCACCCACAATCTCTCA[A/T]TT

>SNP_51

TGCAGTCAC[A/C]TGGAAACAACAAAGAACGAAGGATCCGAAGGGTAACAAACAATTTGGCCAAACAGAAAC

>SNP_52

TGCAGACCTAAA[A/G]ATAGCAAGTTTGACATATTTTCCTTACAGATCGGAAGAGCGGTTCAGCAGGAATGC

>SNP_53

TGCAGAGTGGTGTTTTTTGGAGTCAGTGTTATTGGAGCTTGGTTTCCCTGAAGTTTGTGTTAGATG[A/G]AT

>SNP_54

TGCAGTGTAAGTGAAGCAC[C/T]CTCATCTTCTATATTGATCTTGGTTACAGATCGGAAGAGCGGTTCAGCA

>SNP_55

TGCAGAT[G/A]CAAGAGGAATAATTGAGTACTGGAATCCCATGACACTTGATTTTCCAGAAAGTGGGTATGT

>SNP_7

TGCAGAGTGTACTT[G/C]TTCTCCCAACTGCGGGTATCGAATCCATCAGATACATGCCGCAAATATGTTAGG

>3644978|F|0--31:G>A

TGCAGCCTAGTATCTAAAAGAAAGGATGGTGGCTATCAAGAAGCATACACACTTGGGGAAAGAGAATGT

>SNP_8

TGCAGCTATAATTGCTATTGTCTATATGGTTTGGAGGGAAAGGAACAATGT[C/A]TTATGGAACCAACAAAT

>SNP_9

TGCAGGACAAGCCTACC[A/T]AGAAATGATGGAATGGTGACATTGGTGGATGAAGTAGGGGAGGAATATCCA

>SNP_17

TGCAGGATA[C/T]GCTCTTGATTTTTTGGTAAATGCATACTGCAACAGAGGCCGAGCTTCTGAAGCTTGCAA

>SNP_18

TGCAGG[T/G]ATTCGAATTGGTTTTGGTTCTATAGCCAGACTGTGTGCAACATTTATACTGCTGTTTAGCAC

>SNP_19

TGCAGTCAACAAGCTTAGGAAAGGCCACC[A/G]ATCTCCTCTTTCAATGAAATGCTCCCTAAAATGGCTAAT

>SNP_4

TGCAGTCACTGTTAGAAGAGGTAGCCATAGCATAATTTTATATATTTTCTACTTCCAAG[C/T]TCTAAAAAA

>SNP_20

TGCAGTCATTCTCTTCCTGTAACTTCTTGTCAGGAAACATAACACTGAACTCA[C/A]TGTAAAATCAGAGCT

>SNP_10

TGCAGTC[C/T]TTCACTGATGCTAAACAAAGGGGGCATACCCATTTCAACATCCAACCCCAAATAGGACCTG

>SNP_1

TGCAGTTTAGAATTTTACAATTTTTTTGTTGTATGTGGAAGTAGCTCATTCACCCACAATCTCTCA[A/T]TT

>SNP_11

TGCAGTCTGAACTTTTCAAAGCTTTTGAGAC[A/G]ACAAGGTTATTACAGATCGGAAGAGCGGTTCAGCAGG

>SNP_56

TGCAGAAACAGTGCTTTGATCTTACTCGTAAGCTCTCTGAACAGGTTTTGAAGACAGAGGAATTCAAGA

>SNP_3

TGCAGA[T/A]ACAGTTAGCGACTAATGATTATACAAACTCCAGTATAGTGCCCCTATATCACCTTTAGATTA

>SNP_13

TGCAGACAATGAACATGTAGGGTAAAATAAACAATGTCATGGATGGACATGG[A/G]TCTTCCACAAATCACT

>SNP_5

TGCAGACTAAACCACTTCATCTG[C/A]AACCTGAACCAGAAGTCCAAAACTCAAAGTGGCTATCGAACATAT

> SNP14/SNP_36780842

TGCAGTAAATAAGCACCTTTGTGGGCCTGTGGCAGTGCTTGAGGCAACTGATGTCAATGA[C/G]TATGAGGT

> SNP21/SNP_48607347

TGCAGTCTTAGTTTCCAACATCTAGAGCTCCAGAAGCAT[T/G]CTCCTCTAAGAGAGGGGGGGCCTTAGAGT

>SNP_15

TGCAGACTTATTGGAAGTAAGGTGGACAACTAACCTCCGTACT[A/G]TTACAGATCGGAAGAGCGGTTCAGC

>SNP_6

TGCAGCTGAACGGGAGAGCATGGCAGTTTGTGTCACCTATATG[A/G]AGGAGGAAACAGAATCAGATATTAC

>SNP_16

TGCAGAATCGCAAAGCAAAGAGGGCGATACTTTCTTTCTCAT[C/T]ACTCAAAGGTTCGTCCTTCCTCCACC

**Supplementary Text 2: R code to compute fishers test on genotype count matrix**

#Definition of Fischer test function

row_fisher <- function(row, alt = 'two.sided', cnf = 0.95) {

+ f <- fisher.test(matrix(row,nrow = 3), alternative = alt, conf.level = cnf)

+ return(c(row,

+ p_val = f$p.value,

+ or = f$estimate[[1]],

+ or_ll = f$conf.int[1],

+ or_ul = f$conf.int[2]))}

df=read.table(“filename”,header=T) #Read genotype count matrix into R

p <- data.frame(t(apply(df, 1, row_fisher))) #Compute the fisher test on the entire matrix

head(p) #Visualise the p value results for a part of the matrix

X1.1 X1.0 X0.1 X1.1.1 X1.0.1 X0.1.1 p_val

1 5 15 36 1 67 42 1.660776e-05

2 1 43 12 2 42 66 2.887532e-06

3 1 12 43 2 66 42 2.887532e-06

4 1 22 33 2 63 45 8.022201e-02

5 0 12 44 0 67 43 1.381785e-06

6 0 11 45 0 68 42 2.250328e-07

write.csv(“p”,sep=\t”) #Store the results in a file
